# Sternectomy Reconstruction Using a Customized Three-Dimensional-Printed Polyetheretherketone Implant

**DOI:** 10.7759/cureus.47216

**Published:** 2023-10-17

**Authors:** Weston A Terrasse, Ahmed Mansour, Marshall G Miles, Timothy Misselbeck, Randolph Wojcik

**Affiliations:** 1 Division of Plastic and Reconstructive Surgery, Lehigh Valley Health Network, Allentown, USA; 2 Cardiac Surgery, University of South Florida Morsani College of Medicine, Allentown, USA

**Keywords:** 3d printed implant, sterno-pectoral region muscles, sternal reconstruction, peek implants, chest wall repair & reconstruction

## Abstract

Complex sternal and chest wall reconstruction can be a challenging clinical situation, with the main objectives being restoration of chest wall rigidity, protection of intrathoracic organs, preservation of respiratory function, and reduction of pain and clicking. The treatment of choice is varied, with several different materials available to aid in adequate reconstruction. We present the case of a 60-year-old male with a post-sternectomy defect and debilitating symptoms who underwent reconstruction with a customized, three-dimensional (3D)-printed polyetheretherketone (PEEK) implant and pectoralis muscle flaps. There were no complications in the perioperative period, and the patient reported significant improvement in pain and subjective improvement in chest stability and respiration. The use of PEEK as a reconstructive material for cardiothoracic defects is a viable and safe method that has several important benefits over other utilized materials in the literature. The early success of this case in relieving patient symptoms opens the door for further exploration of PEEK as an alternative for cardiothoracic reconstruction.

## Introduction

Complex chest wall and sternal reconstruction can be a challenging clinical situation, often requiring a multidisciplinary approach and innovative preoperative planning. The primary goals of such a procedure include restoration of chest wall rigidity, protection of intrathoracic organs, preservation of pulmonary function, reduction of pain and clicking, adequate soft tissue coverage, and esthetic considerations [1]. 

Several biological, synthetic, and metallic materials are available to reconstruct the chest wall, including titanium plating, methyl methacrylate, prosthetic patches, and bone allografts and autografts [2]. Polyetheretherketone (PEEK) is a thermoplastic polymer that presents a number of benefits over previously used modalities, namely its analogous properties with cortical bone, radiolucency, MRI compatibility, ability to be crafted with a three-dimensional (3D) printer, and lack of donor site morbidity [2,3].

While there is extensive reporting on the use of PEEK implants in various orthopedic, neurosurgical, and maxillary surgery literature, there is minimal documentation of the application of PEEK in the cardiothoracic and sternal reconstruction realms. Here, we present a case of a patient presenting with debilitating pain and clicking resulting from a sternectomy. The patient was treated with a unique team approach between cardiothoracic and plastic surgery, using a custom 3D-printed PEEK sternal implant and pectoralis muscle flaps.

## Case presentation

A 60-year-old male with a medical history of coronary artery disease, hyperlipidemia, hypertension, and chronic obstructive pulmonary disease presented to the thoracic surgery office in December 2022 for consideration for sternal reconstruction. The patient had a coronary artery bypass graft in August 2021 that subsequently developed sternal dehiscence. This was further complicated by the progression to infection that ultimately necessitated debridement and sternectomy in February 2022, all of which was done at an outside hospital. Afterward, the patient complained of sternal pain, clicking, and respiratory distress affecting his quality of life. Following examination by both the cardiothoracic and plastic surgery teams, the decision was made to pursue sternal reconstruction in May of 2023 with a custom 3D-printed PEEK sternal implant in a combined effort.

**Figure 1:**
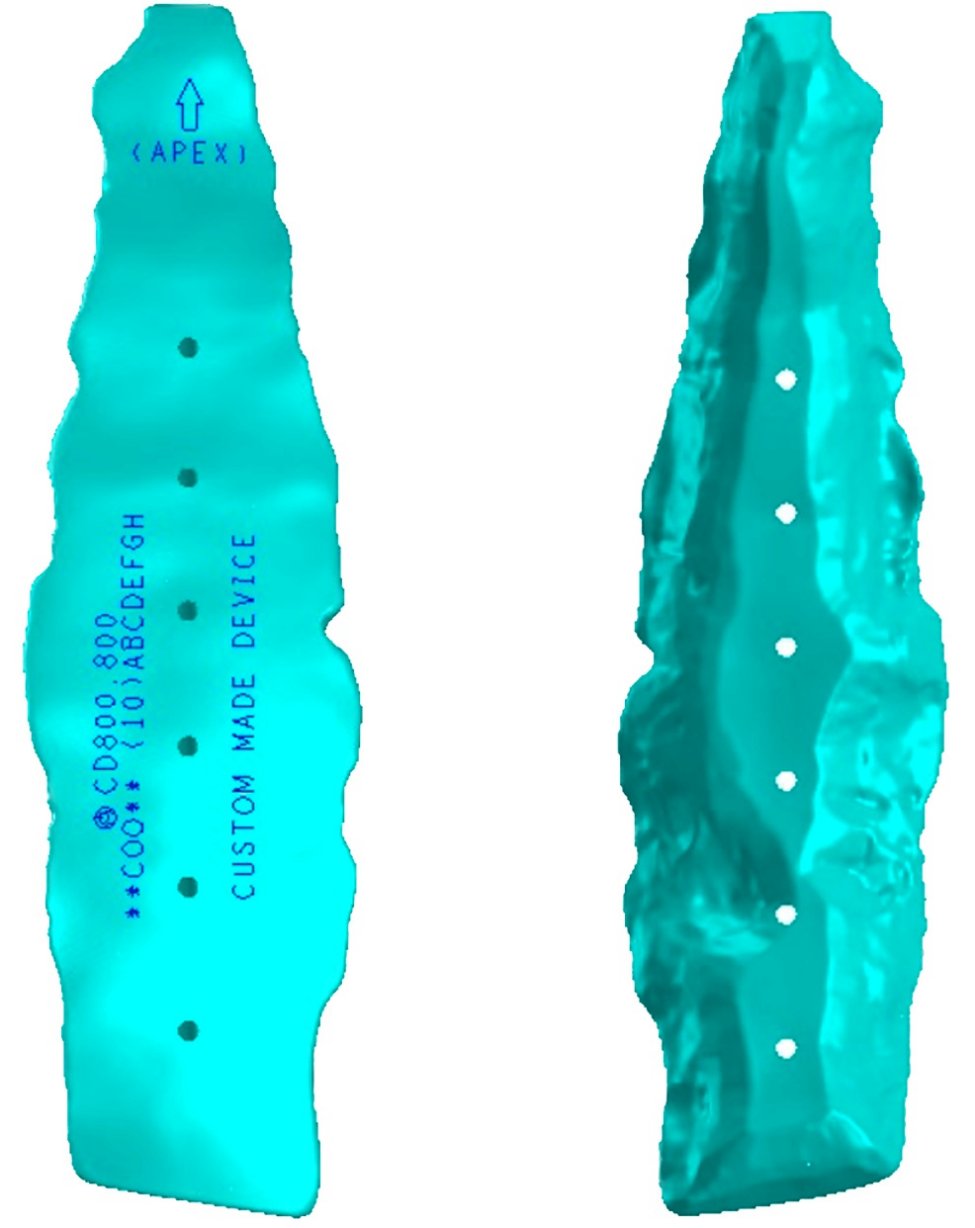
Custom PEEK sternum fixation implant (anterior face, left; posterior face, right). PEEK: Polyetheretherketone.

**Figure 2:**
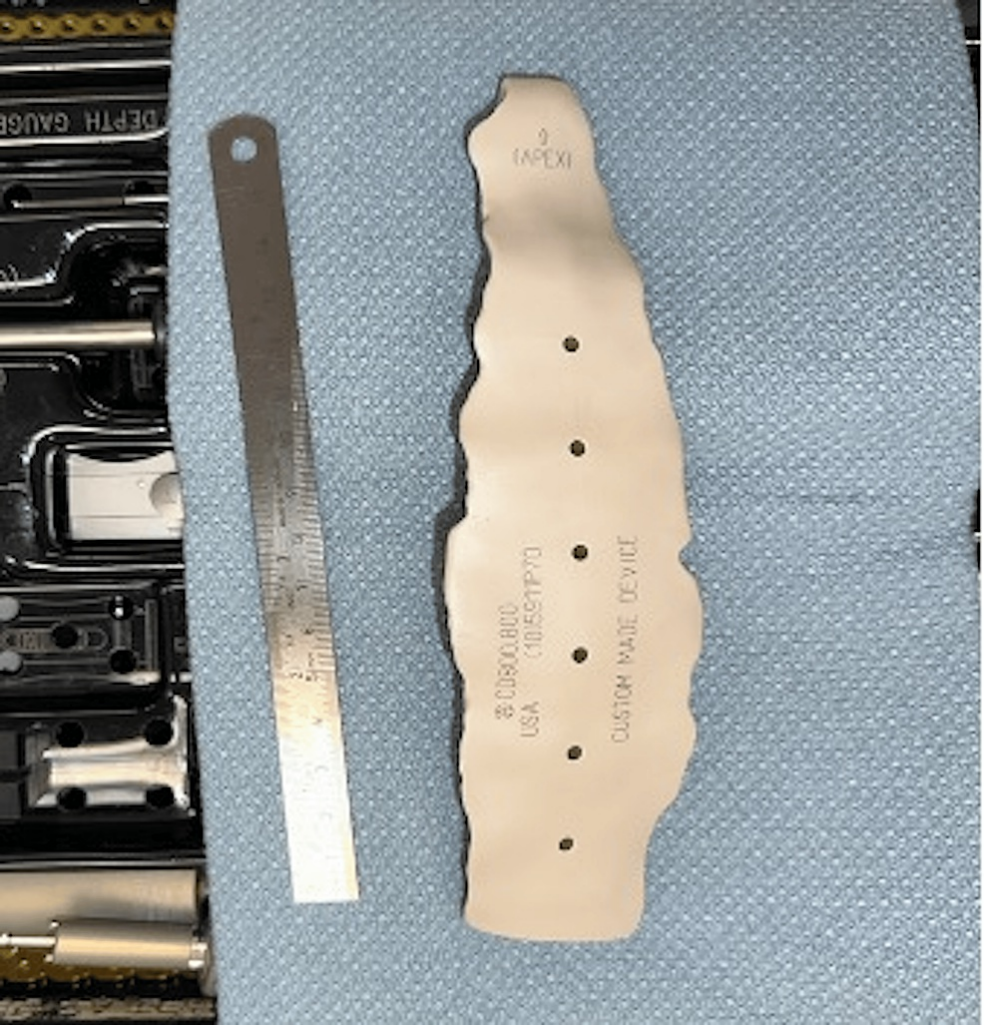
3D-printed PEEK sternum implant (17 x 4 cm) prior to surgical implantation. PEEK: Polyetheretherketone.

Image files of the patient’s relevant anatomy were sent to a medical device company with a comprehensive orthopedics portfolio, which produced a custom implant using 3D processing software (Figures 1, 2). PEEK material was chosen because its mechanical properties are most like the bony tissue being replaced. 

**Figure 3:**
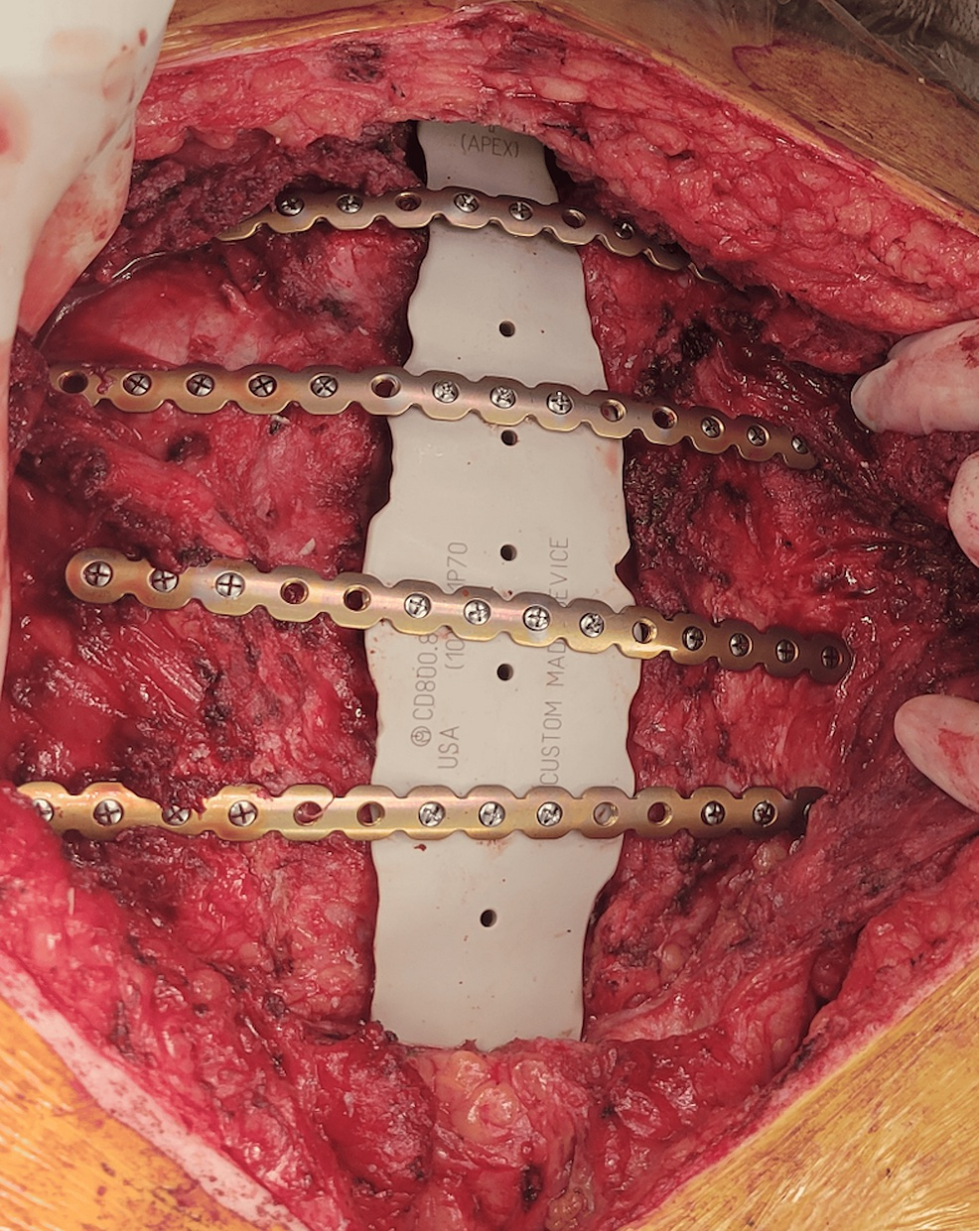
PEEK implant with plates secured bilaterally on ribs one through five. PEEK: Polyetheretherketone.

Intraoperatively, the full extent of the patient’s sternal soft tissue region was resected to remove old scar tissue, subcutaneous tissue, and some fascia. There was no evidence of an infection. The right pectoralis major muscle flap was raised, followed by re-elevation of the left pectoralis major, which had been utilized previously as a turnover flap. The sternal implant was then placed with an appropriate fit, and four titanium plates were fashioned and secured widely to the rib edges and the costochondral junctions. The plates were secured bilaterally to the implant and ribs one through five (Figure 3). Plastic surgery completed the final portion of the case by insetting both pectoral muscles as advancement flaps over the PEEK implant and placing two 15 French drains. The hardware was covered with minimal tension and well-perfused muscle flap tissue and skin coverage. 

The patient had an uncomplicated postoperative course and remained inpatient until postoperative day three for appropriate analgesia and evaluation by physical therapy prior to discharge. While admitted, he received 24 hours of perioperative cefazolin, deep vein thrombosis (DVT) prophylaxis with subcutaneous heparin, and pain control with scheduled Tylenol, as well as oxycodone and methocarbamol as needed. He presented in the outpatient setting three and five weeks postoperatively with a well-healed sternal incision and no evidence of infection or dehiscence. The patient reported significant improvement in pain and subjective improvement in chest stability and respiration.

## Discussion

Surgical planning for complex chest wall and sternal reconstruction can be a challenging task, and here we present a successful, innovative approach using a custom 3D-printed PEEK implant. PEEK materials have good biocompatibility and stability as a suitable bone substitute, have radiographic translucency and produce no artifacts on radiographic imaging [4]. One important drawback to note is the increased cost of PEEK material compared with other synthetic substitutes or autologous tissue. A balance between sufficient rigidity to prevent paradoxical breathing while avoiding too much rigidity that may impair chest wall motion is an important consideration in these cases. While titanium and methyl methacrylate prostheses were the most commonly used materials in the past, PEEK presents a lighter and more flexible option for reconstruction that is also sturdier and more similar to bone than various forms of mesh [5,6].

## Conclusions

The treatment of complex sternal defects is a challenging, often multidisciplinary surgical issue. Several different materials are available to aid in adequate reconstruction, and here we present the case of a patient who underwent sternal reconstruction with a customized, 3D-printed PEEK implant and muscle flaps. One limitation of the study is the short-term patient follow-up results, given the recency of the surgery. Long-term evaluation will be necessary to determine the efficacy of the implant, in addition to comparing outcomes with those of a cohort of individuals who have undergone the same procedure. The early success of this case in relieving patient symptoms opens the door for further exploration of PEEK as an alternative for cardiothoracic reconstruction. 
